# Case Report: Unveiling segmental hemodynamic heterogeneity in internal jugular vein stenosis: a patient-specific CFD analysis

**DOI:** 10.3389/fcvm.2025.1681287

**Published:** 2026-03-24

**Authors:** Hui Li, Jian Dong, Chunxiao Lu, Xiao Xue, Lu Liu, Weiyue Zhang, Yifan Zhou, Huimin Jiang, Yali Wu, Beibei Mao, Guangtong Zhu, Haiyang Ma, Jian Chen, Zhiqiang Hu, Chen Zhou, Xunming Ji

**Affiliations:** 1 Department of Neurology, Xuanwu Hospital, Capital Medical University, Beijing, China; 2 Neuroscience Center, Beijing Shijitan Hospital, Capital Medical University, Beijing, China; 3 Department of Radiology, Beijing Tiantan Hospital, Capital Medical University, Beijing, China; 4 Ophthalmology Department, Xuanwu Hospital, Capital Medical University, Beijing, China; 5 Beijing Advanced Innovation Center for Big Data-Based Precision Medicine, School of Biological Science and Medical Engineering, Beihang University, Beijing, China; 6 Beijing Institute of Brain Disorders, Laboratory of Brain Disorders, Ministry of Science and Technology, Collaborative Innovation Center for Brain Disorders, Beijing Advanced Innovation Center for Big Data-based Precision Medicine, Capital Medical University, Beijing, China; 7 Department of Neurosurgery, Beijing Shijitan Hospital, Capital Medical University, Beijing, China; 8 Department of Neurosurgery, Xuanwu Hospital, Capital Medical University, Beijing, China

**Keywords:** internal jugular vein stenosis, cerebral venous outflow insufficiency, internal jugular vein decompression, extrinsic venous compression, segmental venous stenosis

## Abstract

**Background and purpose:**

While non-thrombotic internal jugular vein stenosis (IJVS), predominantly from extrinsic compression, is increasingly linked to cerebral venous outflow insufficiency, its segment-specific pathophysiology remains unclear. Given the distinct J1-J3 anatomy of the internal jugular vein, segment-based characterization may aid management.

**Case presentation:**

We retrospectively analyzed five patients with non-thrombotic IJVS affecting different segments and etiologies. Clinical features, segment involvement, and lesion morphology were recorded. Patient-specific hemodynamics were modeled with computational fluid dynamics (CFD) from head-neck CT venography (CTV) using identical boundary conditions for cross-case comparison. All cases showed trans-stenotic pressure gradients that increased with stenosis severity. Elevated wall shear stress localized to the stenosis, with high-velocity jets within the narrowed segment and downstream vortices in post-stenotic regions. J3 stenoses from C1 transverse process and/or styloid compression improved after decompression, with better hemodynamic metrics, venous morphology, and symptoms. J1-J2 stenoses were mainly due to soft-tissue or arterial compression; feasible interventions were limited and conservative care yielded modest benefit.

**Conclusions:**

IJVS demonstrates segment-specific morphological and hemodynamic patterns. Patient-specific CFD derived from CTV quantitatively characterizes these abnormalities and may inform treatment selection and prognosis, although validation in larger cohorts is warranted.

## 1 Background

Cerebral venous outflow insufficiency (CVOI) is an under-recognized subset of cerebrovascular disease. Internal jugular vein stenosis (IJVS) is a leading cause of CVOI and has been linked to chronic, treatment-refractory symptoms, including dizziness, headache, visual impairment, tinnitus, tinnitus cerebri, subjective hearing loss, emotional abnormality, memory loss, sleep disorder, and neck discomfort, substantially impacting quality of life (1–6). Compared with the carotid artery, the internal jugular vein's (IJV) thin wall and limited smooth muscle make it vulnerable to extrinsic compression, most commonly from bone, and less often from muscles, arteries, or lymph nodes (3, 7–14). Anatomically, the IJV comprises three segments with distinct characteristics and stenotic mechanisms: J1, from the brachiocephalic confluence to the superior thyroid vein inflow; J2, from the superior thyroid vein to the common carotid bifurcation; and J3, from the carotid bifurcation to the skull base (jugular foramen) (2, 13).

Within this segment-based framework, we report five IJVS cases spanning different segments and etiologies, summarize anatomical patterns and putative compressive mechanisms with imaging correlates, and use patient-specific computational fluid dynamics (CFD) from head-and-neck CT venography (CTV) (under uniform boundary conditions) to compare segmental hemodynamics. These data provide preliminary guidance for segment-informed diagnosis and management and motivate larger studies.

## 2 Materials and methods

### 2.1 Study design and participants

We retrospectively included five patients with IJVS involving different venous segments and etiologies. The study was approved by the Ethics Committee of Beijing Shijitan Hospital, Capital Medical University (sjtkyl1-1x-2022(013)) and conducted in accordance with the Declaration of Helsinki. Written informed consent was obtained from all patients for publication of the case series and images. All patients underwent contrast-enhanced head-neck CTV. Clinical records and imaging were independently reviewed by two experienced neuroradiologists, who reached a consensus IJVS diagnosis. For each patient, we recorded presenting symptoms, involved IJV segment(s), lesion morphology, treatment, and outcomes.

### 2.2 CFD workflow

Patient-specific CFD was performed using CTV-derived geometries and identical boundary conditions across cases. Three-dimensional reconstructions were created in Materialise Mimics 21, smoothed and meshed in 3-matic 13.0, and simulated in Ansys 2022. Following prior methods, vessel walls were treated as rigid; blood was modeled as an incompressible Newtonian fluid with laminar flow, density 1,050 kg/m³, and dynamic viscosity 0.0035 Pa·s (15). No-slip conditions were applied at walls, and outlet pressure was fixed at 0 cmH₂O at model exits (15). Outputs included wall shear stress, intraluminal pressure, velocity fields, and etc.

## 3 Case presentation

### 3.1 Case 1: J1 segment stenosis

**Figure 1:**
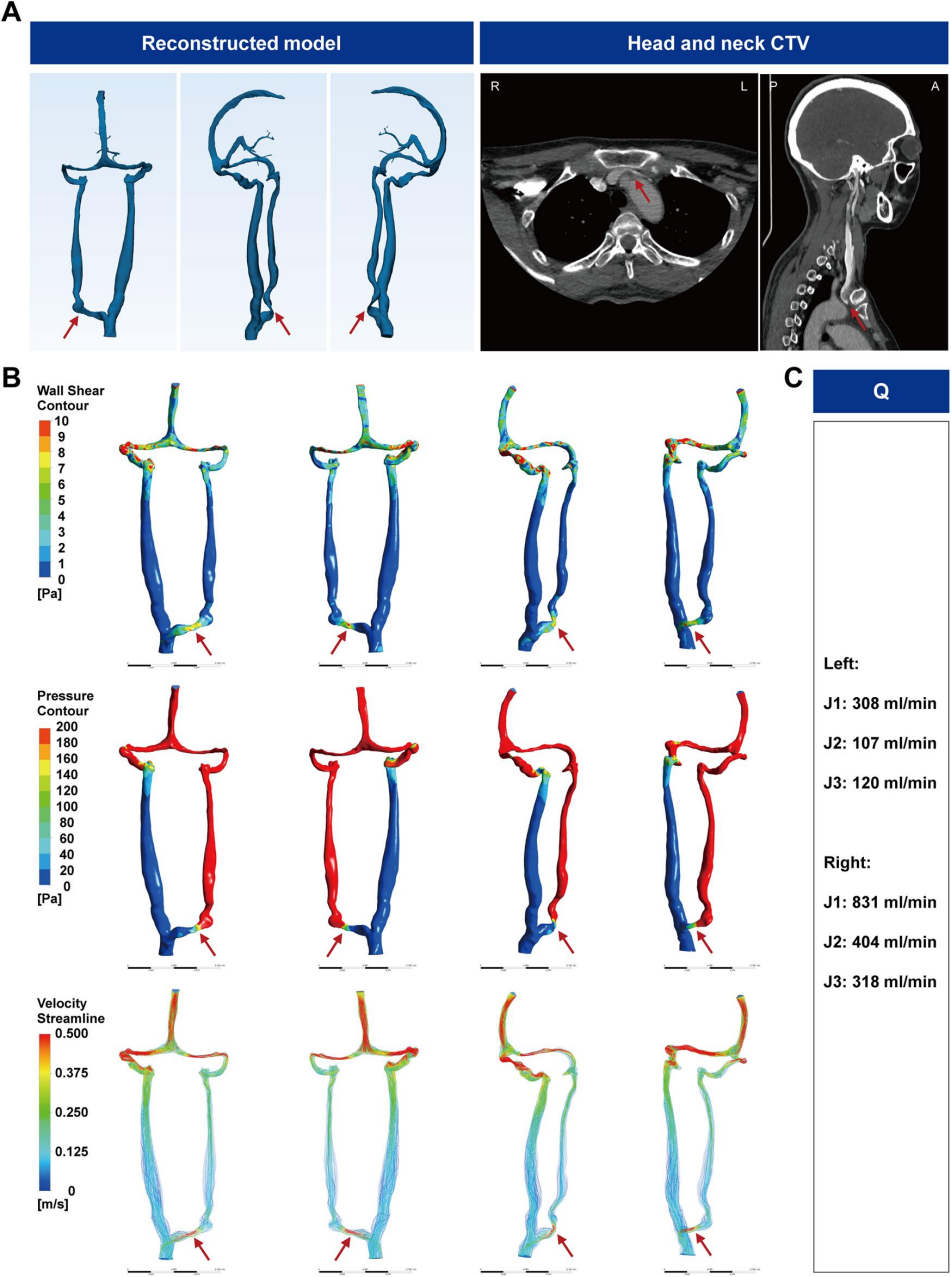
CTV images of the head and neck of IJVS in the J1 segment. **(A)** Reconstructed three-dimensional CTV images of the head and neck, with axial, sagittal, and coronal views showing compression of the left J1 segment IJV by the sternum and aorta; **(B)** Hemodynamic analysis results based on CFD; **(C)** Bilateral IJV blood flow measurements by the 3D-JVFI. CTV, CT venography; IJVS, internal jugular vein stenosis; IJV, internal jugular vein; CFD, computational fluid dynamics; Q, blood flow; 3D-JVFI, three-dimensional jugular venous flow imaging.

A 53-year-old woman had 2 years of persistent whole-head tinnitus with binocular blurring and poor sleep. Head-neck CTV showed the left J1/proximal IJV coursing between the aorta and sternum with extrinsic compression and luminal flattening (Figure 1A). Three-dimensional jugular venous flow imaging (3D-JVFI) demonstrated reduced left-sided jugular flow. CFD (Supplementary Table 1 and Figure 1B) identified a minimum cross-sectional area (SCA) of 1.23 × 10^−^⁵ m², consistent with 82.14% narrowing, and a mild trans-stenotic pressure gradient (1.88 mmHg). Simulations showed post-stenotic velocity acceleration with localized WSS elevation and intraluminal pressure drop (jet effect). Integrating clinical and imaging/CFD findings, we diagnosed left-sided J1 IJVS due to aorta-sternum compression. Stenting/decompression was unsuitable; surveillance was advised. At 3 months, symptoms were unchanged.

### 3.2 Case 2: J2 segment stenosis

**Figure 2:**
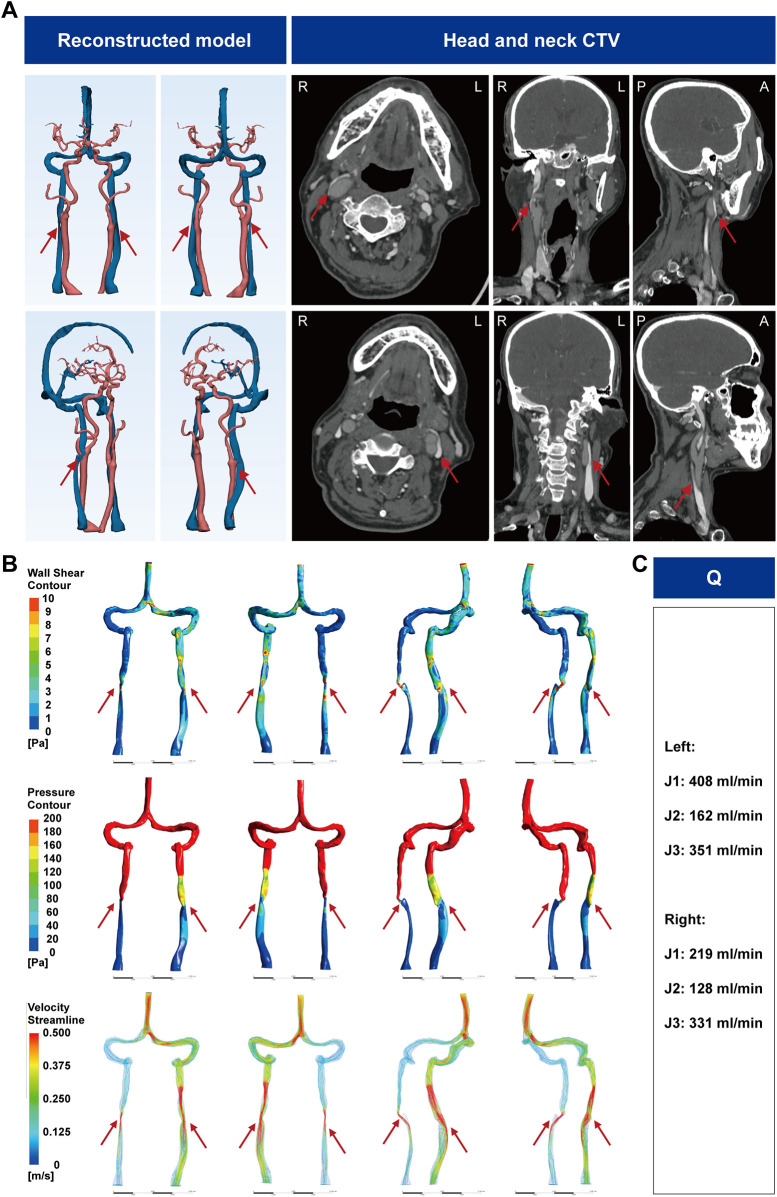
CTV images of the head and neck of IJVS in the J2 segment. **(A)** Reconstructed three-dimensional CTV images of the head and neck, with axial, sagittal, and coronal views showing bilateral J2 segment IJV compression by common carotid artery atherosclerotic plaques; **(B)** Hemodynamic analysis results based on CFD; **(C)** Bilateral IJV blood flow measurements by the 3D-JVFI. CTV, CT venography; IJVS, internal jugular vein stenosis; IJV, internal jugular vein; CFD, computational fluid dynamics; Q, blood flow; 3D-JVFI, three-dimensional jugular venous flow imaging.

A 70-year-old man had 6 months of persistent whole-head tinnitus. Head-neck CTV showed bilateral J2-segment IJV compression between the sternocleidomastoid (SCM) and common carotid artery (CCA) with luminal flattening (Figure 2A). 3D-JVFI demonstrated reduced bilateral IJV flow, greater on the right. CFD (Supplementary Table 2 and Figure 2B) found a minimal cross-sectional area of 4.84 × 10^−^⁶ m² (85.45% stenosis) on the right and 3.00 × 10^−^⁵ m² (59.10%) on the left, with post-stenotic velocity acceleration and flow disturbance; right-sided mean velocity increased more, whereas left-sided vortical features were more conspicuous. Proximal J2 segments showed localized WSS elevation and mural pressure drop, less marked on the left; the trans-stenotic pressure gradient was 5.08 mmHg (right) vs. 0.76 mmHg (left). Integrating clinical and hemodynamic data, we diagnosed bilateral J2 IJVS due to extrinsic SCM-CCA compression. As stenting/decompression was unsuitable and no effective medical therapy exists, we recommended surveillance; symptoms were unchanged at 3 months.

### 3.3 Case 3: bilateral J3 segment stenosis—C1 transverse process compression

**Figure 3:**
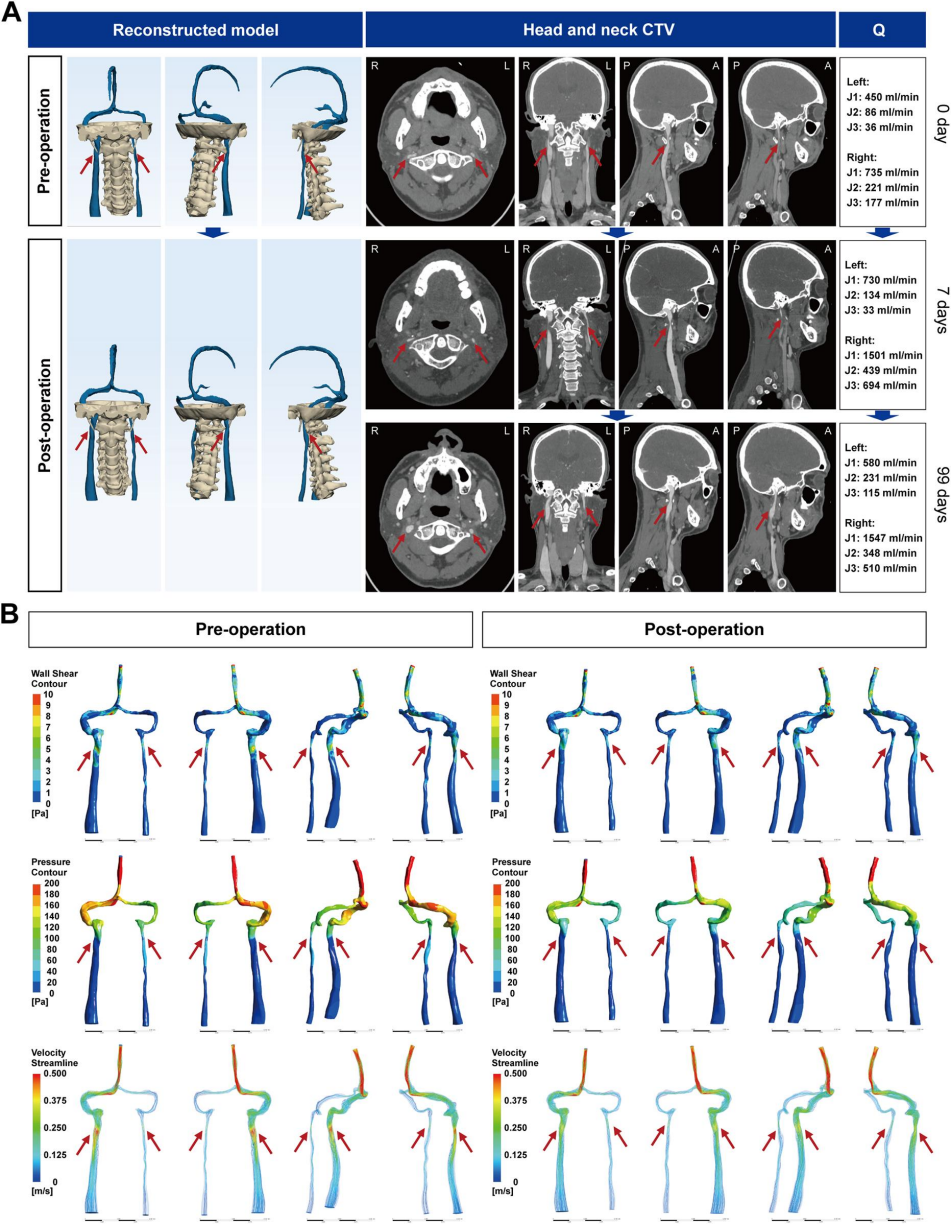
CTV images of the head and neck of IJVS in the J3 segment. **(A)** Reconstructed three-dimensional CTV images of the head and neck before and after IJV decompression surgery, with axial, sagittal, and coronal views showing bilateral J3 segment IJV compression by the C1 transverse process. Postoperative images at day 7 and day 99 demonstrate progressive IJV expansion, along with bilateral IJV blood flow measurements; **(B)** Hemodynamic analysis results based on CFD before and after IJV decompression surgery. CTV, CT venography; IJVS, internal jugular vein stenosis; IJV, internal jugular vein; CFD, computational fluid dynamics; Q, blood flow.

A 33-year-old man had 1 month of intermittent dizziness, blurred vision, and episodic bilateral tinnitus. Head-neck CTV showed right-dominant drainage with bilateral J3 IJV stenosis at the C1 transverse processes and posterior cervical collaterals (Figure 3A). 3D-JVFI confirmed reduced bilateral jugular flow. CFD (Supplementary Table 3 and Figure 3B) found minimal cross-sectional areas of 2.96 × 10^−^⁶ m² (right) and 5.28 × 10^−^⁶ m² (left), corresponding to 63.7% and 61.7% stenoses, with low trans-stenotic pressure gradients (0.75 and 0.47 mmHg). Simulations showed post-stenotic jetting with focal WSS elevation and pressure drop, greater on the right. Preoperative ophthalmology showed no papilledema (Supplementary Figure 1). On November 24, 2022, bilateral IJV decompression was performed via partial resection of the C1 transverse processes (6 mm right, 7 mm left). Early postoperative imaging demonstrated decreased stenosis and increased jugular flow with mild symptom improvement. By day 99, dizziness, blurred vision, and tinnitus had completely resolved. CTV showed substantial relief of IJV compression with adequate venous opacification, and 3D-JVFI confirmed further flow improvement. Postoperative CFD demonstrated lower segmental mean velocity at J3 with attenuated WSS elevations and pressure drops vs. baseline.

### 3.4 Case 4: unilateral J3 stenosis—C1 transverse process compression

**Figure 4:**
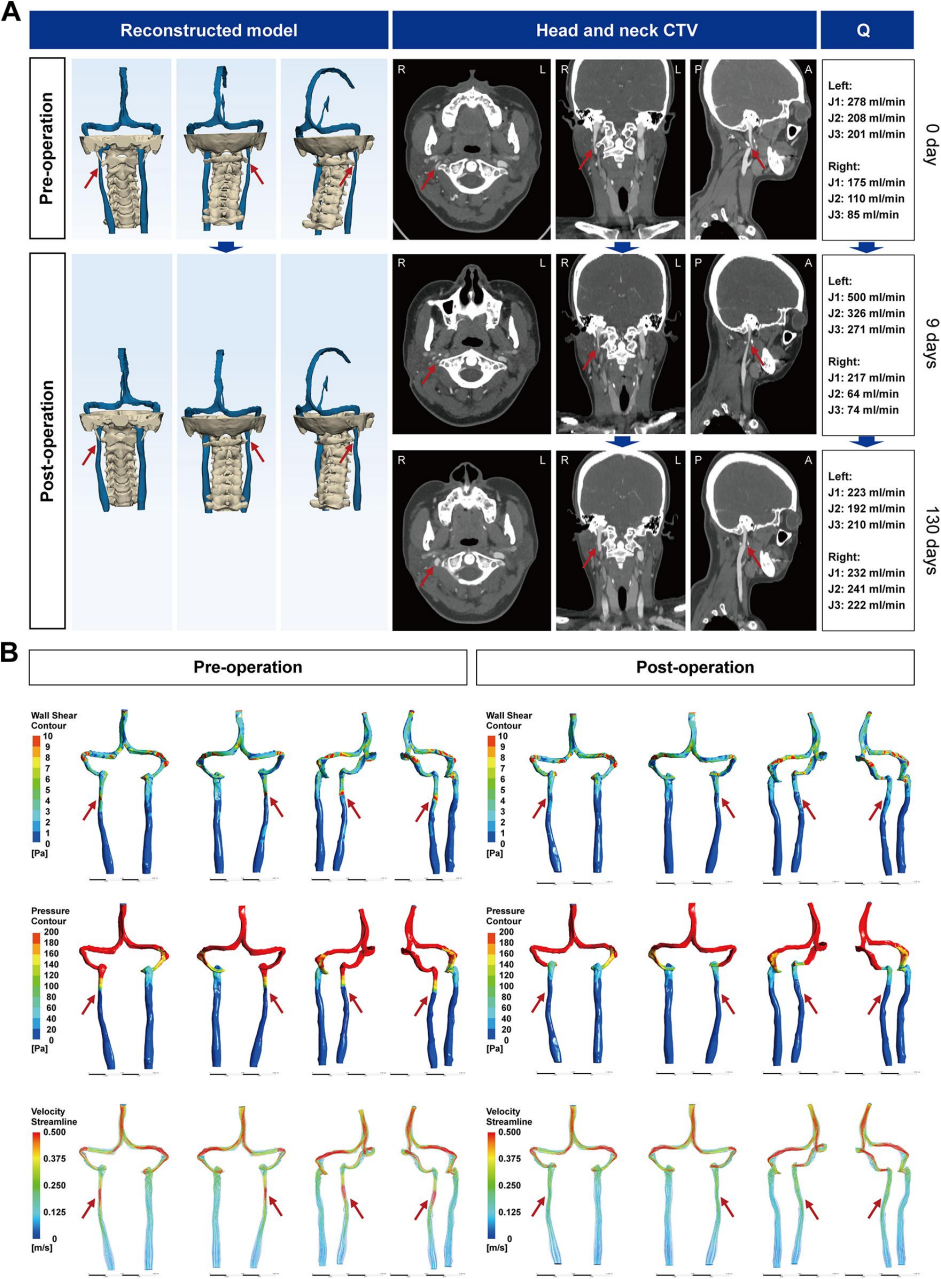
CTV images of the head and neck of IJVS in the J3 segment. **(A)** Reconstructed three-dimensional CTV images of the head and neck before and after IJV decompression surgery, with axial, sagittal, and coronal views showing J3 segment IJV compression by the right C1 transverse process. Postoperative images at day 9 and day 130 demonstrate progressive IJV expansion, along with bilateral IJV blood flow measurements; **(B)** Hemodynamic analysis results based on CFD before and after IJV decompression surgery. CTV, CT venography; IJVS, internal jugular vein stenosis; IJV, internal jugular vein; CFD, computational fluid dynamics; Q, blood flow.

A 33-year-old woman had intermittent dizziness and headache for 10 years, 1 year of persistent tinnitus, cerebri tinnitus, and mild bilateral hearing loss. Head-neck CTV showed right J3 IJV stenosis at the C1 transverse process with linear narrowing (Figure 4A). CFD (Supplementary Table 4 and Figure 4B) demonstrated a minimal cross-sectional area of 1.16 × 10^−5^ m² (61.30% narrowing), post-stenotic jetting with WSS elevation and pressure drop, and a modest trans-stenotic gradient (1.68 mmHg). 3D-JVFI confirmed reduced right IJV flow. A diagnosis of right-sided J3 IJVS was made. On November 25, 2020, the patient underwent right IJV decompression with approximately 4 mm resection of the C1 lateral mass. Early postoperative course showed symptomatic improvement; on postoperative day 9, CTV demonstrated partial C1 resection with mild soft-tissue swelling/residual air and only mild reduction of stenosis, and 3D-JVFI did not show a clear flow increase, producing a temporary compensatory increase in left IJV flow. Then, the patient was discharged in stable condition. At 130 days, there was marked relief of dizziness, headache, tinnitus, and cerebri tinnitus (hearing loss unchanged). CTV showed resolution of swelling/air and substantial IJV luminal expansion with improved opacification; 3D-JVFI confirmed increased right IJV flow. Postoperative CFD indicated lower segmental mean velocity with attenuated WSS elevations and pressure drops vs. baseline.

### 3.5 Case 5: unilateral J3 stenosis—C1 and styloid process compression

A 36-year-old woman had 7 months of persistent cerebri tinnitus that evolved from a mild vertex “electrical buzz” to a high-pitched, cicada-like noise maximal over the right temporo-occipital region, worse at night, with insomnia, anxiety, and subjective memory decline. Otologic disease was excluded. MR venography suggested extrinsic upper right IJV stenosis. Head-neck CTV showed the right J3 segment coursing between the styloid process and the C1 transverse process with focal linear stenosis and tortuous posterior cervical collaterals (Supplementary Figure 2A). 3D-JVFI confirmed reduced right IJV flow. CFD (Supplementary Table 5 and Figure 2B) demonstrated a minimal cross-sectional area of 3.74 × 10^−^⁶ m² (94.39% stenosis), near-interruption of flow with marked post-stenotic jetting and vortical disturbance, pronounced WSS elevation (75.03 Pa), and a mural-pressure drop of 7.35 mmHg. A diagnosis of right-sided J3 IJVS was made, and surgical decompression was recommended. On September 28, 2022, right IJV decompression was performed with about 6 mm resection of the C1 lateral mass. Symptoms did not improve immediately. On postoperative day 2, CTV showed partial C1 resection with mild soft-tissue swelling and minimal residual air; focal atlas-level stenosis persisted but was slightly improved, and 3D-JVFI still showed low right IJV flow. The patient was discharged in stable condition. At day 323, cerebri tinnitus intensity had decreased and sleep/anxiety had improved. Follow-up CTV showed resolution of swelling and air with substantial IJV luminal expansion and no significant residual stenosis; 3D-JVFI confirmed increased right IJV flow. Postoperative CFD indicated lower segmental mean velocity with attenuated WSS elevations and pressure drops vs. baseline.

## 4 Discussion

IJVS, particularly when caused by extrinsic compression, remains a controversial and incompletely characterized vascular entity. Although growing clinical interest has spurred investigation into its hemodynamic consequences and symptom correlations, the IJV is anatomically partitioned into J1, J2, and J3 segments, each with distinct physiological and anatomical attributes. Using a segment-based framework, this study explored segment-specific hemodynamic profiles, morphological features, and clinical outcomes across IJVS subtypes.

In the present series, multi-positional head-and-neck CTV and three-dimensional reconstructions of patient-specific venous anatomy together with adjacent osseous and soft-tissue structures further delineated the pattern of IJVS in J1, J2, and J3 segments. J3 stenosis, the most prevalent subtype, primarily results from osseous compression by the C1 transverse process and/or styloid process (2, 16, 17). CTV and three-dimensional reconstructions confirmed its superficial course adjacent to bone. Unilateral or bilateral posterior IJV compression with collateralized lateral outflow was frequently observed, aligning with prior case-based reports (1, 18). To capture J3 heterogeneity, we defined three imaging subtypes: (1) unilateral C1 transverse process compression, (2) bilateral C1 osseous compression, and (3) combined C1 plus styloid compression. And CFD analyses demonstrated consistent post-stenotic velocity acceleration, flow disturbance, elevated WSS, and reduced mural pressure across three identified radiographic subtypes (unilateral C1, bilateral C1, and combined C1/styloid compression). Surgical decompression (transverse process resection, styloidectomy, or both) effectively improved venous caliber, alleviated symptoms, and normalized hemodynamic parameters, reducing WSS and pressure gradients. Early studies occasionally showed perivenous edema or residual narrowing that limited immediate relief, but by about 3 months further luminal gain and clinical improvement were common. Postoperative CFD at approximately 3 months confirmed sustained hemodynamic improvement, underscoring the need for an adequate recovery period. While surgical outcomes are promising, generalizability remains limited by sample size and follow-up duration.

Across our cohort, J1/J2 cases showed minimal symptom improvement at 3 months. J2 stenosis, located within the carotid sheath, is typically attributable to extrinsic soft-tissue or arterial compression, most commonly the SCM muscle or CCA (13). In our J2 case, CFD demonstrated increased velocity, disturbed flow, elevated WSS, and a substantial right-sided trans-stenotic gradient (5.08 mmHg). Additionally, this series also delineates a proximal (J1) IJVS subtype with clinical, imaging, and hemodynamic features (22). CFD revealed similar hemodynamic disturbances. Usually left-sided and spanning the retrosternal space to the aortic arch, J1 disease reflects mediastinal compression. However, venous decompression at J1/J2 in these patients were not avoided because of surgical inaccessibility in itself, but because of the nature of the venous pathology and the current evidence base. Specifically, most J1/J2 stenoses in our cohort reflect dynamic soft-tissue or arterial apposition (e.g., SCM/CCA at J2; SCM/CCA/aorta/sternum at J1) acting on a thin-walled, highly compliant IJV. In contrast to J3 osseous compression (C1/styloid), for which standardized bony decompression is reproducible and increasingly supported by clinical series, there is no validated, widely adopted venous decompression technique for soft-tissue/arterial etiologies at J1/J2. While isolated case reports describe symptom relief after omohyoid resection, such interventions remain experimental and unvalidated in larger cohorts (10, 11). Importantly, proximal stenoses (J1/J2) may be modulated by systemic factors, including age, BMI, central adiposity (raising intra-abdominal pressure and cranial displacement of the diaphragm/aortic arch), and arterial calcification (reducing compliance), which could exacerbate compression (19–22). These biologically plausible hypotheses warrant prospective validation using standardized hemodynamic assessment and may ultimately inform medical therapies and conservative management strategies. Taken together, these observations support individualized, segment-targeted management and underscore the need for standardized diagnostic criteria and care pathways.

However, this study has several limitations. This study has several limitations. Its retrospective design and small sample size limit statistical power and generalizability. Although we employed CFD-based hemodynamic analyses, prospective clinical validation is needed to confirm their evaluative utility. While our goal was to provide preliminary insights to inform future diagnostic refinements and segment-specific strategies, external validation in larger, multicenter cohorts is essential before broad clinical adoption.

## 5 Conclusion

Using a segment-based framework, this case series highlights the anatomical heterogeneity and hemodynamic impact of IJVS across J1-J3. And the findings support individualized, segment-specific diagnosis and treatment, with surgical decompression considered for confirmed J3 bony compression and CFD leveraged to quantify trans-lesional pressure/flow changes and to inform operative planning and prognostication. In contrast, J1 and J2 disease, typically related to mediastinal, muscular, or arterial compression, lacked feasible surgical options and showed limited short-term symptomatic benefit with conservative management. Nonetheless, the retrospective design, small sample size, and limited follow-up constrain generalizability.

## Data Availability

The datasets presented in this article are not readily available because the raw data underlying this study consist of patient-specific imaging data (DICOM files) and CFD model files that were generated directly from those clinical images. These datasets contain identifiable clinical information and are therefore subject to institutional ethical regulations and data protection policies. However, clinicians or researchers who meet the criteria for accessing confidential medical data may request the relevant materials directly from the corresponding author (Prof. Xunming Ji, jixm@ccmu.edu.cn), subject to institutional approval and a data-sharing agreement.
