# Supplementary material for: Case Report: Unveiling segmental hemodynamic heterogeneity in internal jugular vein stenosis: a patient-specific CFD analysis
Source: Front Cardiovasc Med. 2026 Mar 24;12:1681287. doi: 10.3389/fcvm.2025.1681287 (PMC13053235; doi:10.3389/fcvm.2025.1681287)
Supplement: Supplementary file 1 [file Datasheet1.docx]

**Unveiling Segmental Hemodynamic Heterogeneity in Internal Jugular Vein Stenosis: A Patient-Specific CFD Analysis**

Hui Li¹˒², Jian Dong³, Chunxiao Lu¹, Xiao Xue⁴, Lu Liu¹, Weiyue Zhang⁵, Yifan Zhou⁶, Huimin Jiang⁶, Yali Wu⁷, Beibei Mao⁷, Guangtong Zhu⁷, Haiyang Ma⁷, Jian Chen⁸, Zhiqiang Hu⁷*, Chen Zhou¹˒²˒⁶*, Xunming Ji¹˒²*

1 Department of Neurology, Xuanwu Hospital, Capital Medical University, Beijing, China

2 Neuroscience Center, Beijing Shijitan Hospital, Capital Medical University, Beijing, China

3 Department of Radiology, Beijing Tiantan Hospital, Capital Medical University, Beijing, China

4 Ophthalmology Department, Xuanwu Hospital, Capital Medical University, Beijing, China

5 Beijing Advanced Innovation Center for Big Data-Based Precision Medicine, School of Biological Science and Medical Engineering, Beihang University, Beijing, China

6 Beijing Institute of Brain Disorders, Laboratory of Brain Disorders, Ministry of Science and Technology, Collaborative Innovation Center for Brain Disorders, Beijing Advanced Innovation Center for Big Data-based Precision Medicine, Capital Medical University, Beijing, China

7 Department of Neurosurgery, Beijing Shijitan Hospital, Capital Medical University, Beijing, China

8 Department of Neurosurgery, Xuanwu Hospital, Capital Medical University, Beijing, China

*Corresponding authors:

Xunming Ji

E-mail: [jixm@ccmu.edu.cn](mailto:jixm@ccmu.edu.cn)

Chen Zhou

E-mail: [chenzhou2013abc@163.com](mailto:chenzhou2013abc@163.com)

Zhiqiang Hu

E-mail: [neuro7@163.com](mailto:neuro7@163.com)

**Supplementary Materials**

**Content**

Supplementary table 1. Hemodynamic analysis results based on CFD of IJVS in the J1 segment…3

Supplementary table 2. Hemodynamic analysis results based on CFD of IJVS in the J2 segment..4

Supplementary table 3. Hemodynamic analysis results based on CFD of IJVS in the bilateral J3 segment by C1 transverse process compression……………………………………..……………5

Supplementary table 4. Hemodynamic analysis results based on CFD of IJVS in the unilateral J3 segment by C1 transverse process compression………………………………………….…….…..6

Supplementary table 5. Hemodynamic analysis results based on CFD of IJVS in the unilateral J3 segment by C1 and styloid process compression………..………………………………………7

Supplementary figure 1. Fundoscopy showing normal optic disc with no edema……..…...8

Supplementary Figure 2. CTV images of the head and neck of IJVS in the J3 segment…………9

Supplementary Table 1. Hemodynamic analysis results based on CFD of IJVS in the J1 segment.

| **Types of IJVS** | **J1-IJVS** |
| --- | --- |
| **Location** | Left |
| **Status** | Non-operative |
| **SMV (m/s)** | 0.60 |
| **SCA (m^2^)** | 1.23e-05 |
| **SR (%)** | 82.14 |
| **TPG (mmHg)** | 1.88 |
| **TPR (%)** | 2.01 |
| **MCA (m^2^)** | 9.19e-05 |
| **MVV (m/s)** | 0.54 |
| **NL (m)** | 0.025 |
| **WSS (Pa)** | 9.51 |

Abbreviations: SMV, Stenosis Maximum Velocity; SCA, minimum stenosis cross-sectional area at the stenosis; SR, Stenosis Ratio; TPG, Trans-stenotic Pressure Gradient; TPR, Trans-stenotic Pressure Ratio; MCA：Maximum Cross-sectional Area; MVV, Maximum Vortex Velocity; NL, Narrowing Length; WSS, Wall Shear Stress.

Supplementary Table 2. Hemodynamic analysis results based on CFD of IJVS in the J2 segment.

| **Types of IJVS** | **J2-IJVS** | |
| --- | --- | --- |
| **Location** | Right | Left |
| **Status** | Non-operative | |
| **SMV (m/s)** | 1.09 | 0.75 |
| **SCA (m^2^)** | 4.84e-06 | 0.00003 |
| **SR (%)** | 85.45 | 59.10 |
| **TPG (mmHg)** | 5.08 | 0.76 |
| **TPR (%)** | 0.85 | 18.10 |
| **MCA (m^2^)** | 2.93e-05 | 8.54e-05 |
| **MVV (m/s)** | 0.36 | 0.56 |
| **NL (m)** | 0.018 | 0.01 |
| **WSS (Pa)** | 57.72 | 19.97 |

Abbreviations: SMV, Stenosis Maximum Velocity; SCA, minimum stenosis cross-sectional area at the stenosis; SR, Stenosis Ratio; TPG, Trans-stenotic Pressure Gradient; TPR, Trans-stenotic Pressure Ratio; MCA：Maximum Cross-sectional Area; MVV, Maximum Vortex Velocity; NL, Narrowing Length; WSS, Wall Shear Stress.

Supplementary Table 3. Hemodynamic analysis results based on CFD of IJVS in the bilateral J3 segment by C1 transverse process compression.

| **Types of IJVS** | **Bilateral J3 IJVS-C1 transverse process** | | | |
| --- | --- | --- | --- | --- |
| **Location** | Right | | Left | |
| **Status** | Pre-operation | Post-operation | Pre-operation | Post-operation |
| **SMV (m/s)** | 0.53 | 0.38 | 0.34 | 0.28 |
| **SCA (m^2^)** | 2.96e-06 | 4.56e-05 | 5.28e-06 | 1.10e-05 |
| **SR (%)** | 63.70 | - | 61.70 | - |
| **TPG (mmHg)** | 0.75 | 0.33 | 0.47 | 0.18 |
| **TPR (%)** | 13.90 | 4.02 | 28.20 | 20.90 |
| **MCA (m^2^)** | 8.44e-05 | 8.66e-05 | 1.25e-05 | 2.93e-05 |
| **MVV (m/s)** | 0.36 | 0.26 | 0.23 | 0.21 |
| **NL (m)** | 0.0074 | - | 0.015 | - |
| **WSS (Pa)** | 8.69 | 5.19 | 4.62 | 1.25 |

Abbreviations: SMV, Stenosis Maximum Velocity; SCA, minimum stenosis cross-sectional area at the stenosis; SR, Stenosis Ratio; TPG, Trans-stenotic Pressure Gradient; TPR, Trans-stenotic Pressure Ratio; MCA：Maximum Cross-sectional Area; MVV, Maximum Vortex Velocity; NL, Narrowing Length; WSS, Wall Shear Stress.

Supplementary Table 4. Hemodynamic analysis results based on CFD of IJVS in the unilateral J3 segment by C1 transverse process compression.

| **Types of IJVS** | **Unilateral J3 IJVS-C1 transverse process** | |
| --- | --- | --- |
| **Location** | Right | |
| **Status** | Pre-operation | Post-operation |
| **SMV (m/s)** | 0.74 | 0.30 |
| **SCA (m^2^)** | 1.16e-05 | 3.44e-05 |
| **SR (%)** | 61.30 | - |
| **TPG (mmHg)** | 1.68 | 0.46 |
| **TPR (%)** | 1.11 | 10.1 |
| **MCA (m^2^)** | 4.46e-05 | 4.43e-05 |
| **MVV (m/s)** | 0.43 | 0.29 |
| **NL (m)** | 0.013 | - |
| **WSS (Pa)** | 19.68 | 3.00 |

Abbreviations: SMV, Stenosis Maximum Velocity; SCA, minimum stenosis cross-sectional area at the stenosis; SR, Stenosis Ratio; TPG, Trans-stenotic Pressure Gradient; TPR, Trans-stenotic Pressure Ratio; MCA：Maximum Cross-sectional Area; MVV, Maximum Vortex Velocity; NL, Narrowing Length; WSS, Wall Shear Stress.

Supplementary Table 5. Hemodynamic analysis results based on CFD of IJVS in the unilateral J3 segment by C1 and styloid process compression.

| **Types of IJVS** | **Unilateral J3 IJVS-C1 and styloid process** | |
| --- | --- | --- |
| **Location** | Right | |
| **Status** | Pre-operation | Post-operation |
| **SMV (m/s)** | 1.34 | 0.29 |
| **SCA (m^2^)** | 3.74e-06 | 4.46e-05 |
| **SR (%)** | 94.39 | - |
| **TPG (mmHg)** | 7.35 | 0.49 |
| **TPR (%)** | 0.25 | 15.10 |
| **MCA (m^2^)** | 4.46e-05 | 6.22e-05 |
| **MVV (m/s)** | 0.25 | 0.26 |
| **NL (m)** | 0.016 | - |
| **WSS (Pa)** | 75.03 | 1.33 |

Abbreviations: SMV, Stenosis Maximum Velocity; SCA, minimum stenosis cross-sectional area at the stenosis; SR, Stenosis Ratio; TPG, Trans-stenotic Pressure Gradient; TPR, Trans-stenotic Pressure Ratio; MCA：Maximum Cross-sectional Area; MVV, Maximum Vortex Velocity; NL, Narrowing Length; WSS, Wall Shear Stress.

Supplementary Figure 1


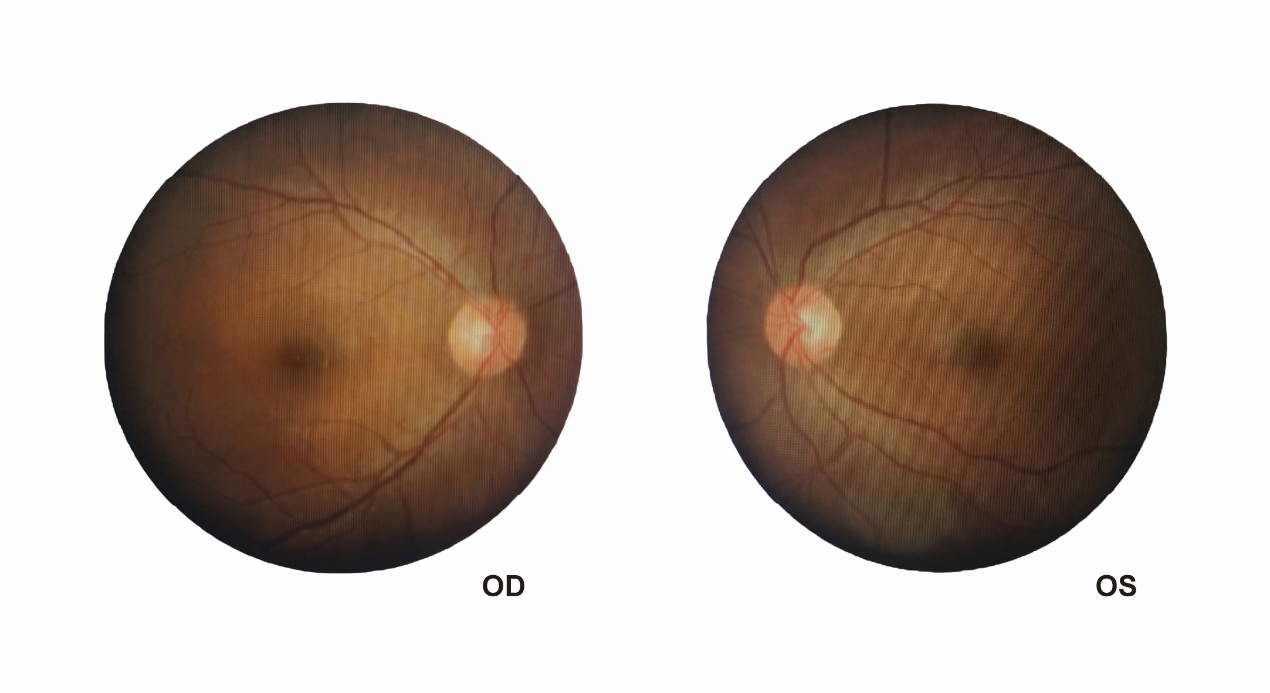
**Supplementary Figure Legends**

Supplementary Figure 1. Fundoscopy showing normal optic disc with no edema. Abbreviations: OD: Oculus Dexte; OS: Oculus Sinister.


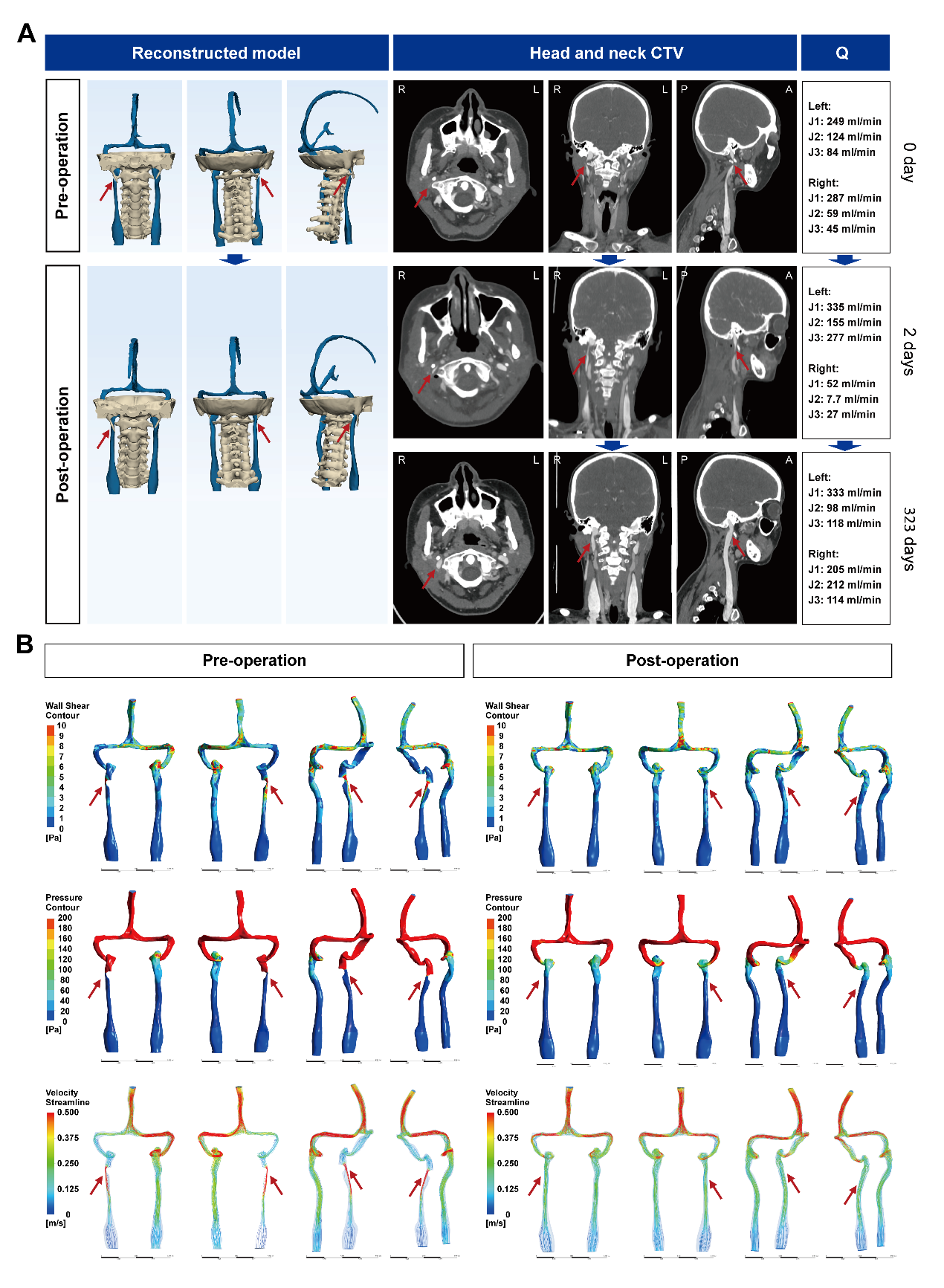


Supplementary Figure 2. CTV images of the head and neck of IJVS in the J3 segment. (A) Reconstructed three-dimensional CTV images of the head and neck before and after IJV decompression surgery, with axial, sagittal, and coronal views showing J3 segment IJV compression by the right C1 transverse process and styloid process. Postoperative images at day 2 and day 323 demonstrate progressive IJV expansion, along with bilateral IJV blood flow measurements; (B) Hemodynamic analysis results based on CFD before and after IJV decompression surgery. Abbreviations: CTV, CT venography; IJVS, internal jugular vein stenosis; IJV, internal jugular vein; CFD, computational fluid dynamics; Q, blood flow.
